# PKA Activates AMPK Through LKB1 Signaling in Follicular Thyroid Cancer

**DOI:** 10.3389/fendo.2019.00769

**Published:** 2019-11-08

**Authors:** Suresh Kari, Vasyl V. Vasko, Shivam Priya, Lawrence S. Kirschner

**Affiliations:** ^1^Department of Cancer Biology and Genetics, The Ohio State University, Columbus, OH, United States; ^2^Uniformed Services University of Health Sciences, Bethesda, MD, United States; ^3^Division of Endocrinology, Diabetes, and Metabolism, Department of Internal Medicine, The Ohio State University, Columbus, OH, United States

**Keywords:** mouse models, thyroid cancer, AMPK, LKB1, PKA

## Abstract

Thyroid cancer affects about one percent of the population, and has seen rising incidence in recent years. Follicular thyroid cancer (FTC) comprises 10–15% of all thyroid cancers. Although FTC is often localized, it can behave aggressively with hematogenous metastasis, leading to an increased risk of cancer death. We previously described a mouse model for FTC caused by tissue-specific ablation of the Protein Kinase A (PKA) regulatory subunit *Prkar1a*, either by itself or in combination with knockout of *Pten*. Loss of *Prkar1a* causes enhanced activity of PKA, whereas ablation of *Pten* causes activation of Akt signaling. At the molecular level, these genetic manipulations caused activation of mTOR signaling, which was also observed in human FTC cases. To understand the mechanism by which PKA activates mTOR, we began by studying intracellular kinases known to modulate mTOR function. Although AMP-activated kinase (AMPK) has been characterized as a negative regulator of mTOR activity, our tumor model exhibited activation of both AMPK and mTOR. To understand the mechanism by which AMPK was turned on, we next studied kinases known to cause its phosphorylation. In this paper, we report that PKA leads to AMPK activation through the LKB1 kinase. Although LKB1 has traditionally been considered a tumor suppressor, our data indicates that it may have a complex role in the thyroid gland, where its activation appears to be frequently associated with follicular thyroid carcinoma in both mice and humans.

## Introduction

Thyroid cancer is the most common endocrine malignancy, affecting about one percent of the population. It is also the cancer that is rising at the fastest rate. Although there appears to be an ascertainment bias, recent data suggests that the increased incidence is also driven by other factors as well (1–3). Of the thyroid cancers, the majority are designated as papillary thyroid cancer (PTC), whereas the next most common type is follicular thyroid cancer (FTC) (4). Although the behavior of FTCs varies widely, this type of cancer has a stronger predilection for hematogenous dissemination (5); once metastatic, there exist no curative therapies for this type of cancer. An enhanced incidence of FTC relative to PTC is associated with Carney complex (CNC) and Cowden syndrome (CS), two tumor predisposition syndromes (6, 7). CNC is caused by inactivating mutations in *PRKAR1A*, encoding for the type 1a regulatory subunit of cAMP (cyclic adenosine monophosphate) dependent protein kinase, or PKA (6); loss of this regulatory subunit causes an increase in PKA activity. CS, on the other hand, is most commonly caused by inactivating mutations of *PTEN*, leading to increased signaling through the PI3K/AKT pathway (7). In order to probe the role of these genes in thyroid cancer, our lab generated mice carrying a thyroid-specific knockout of *Prkar1a* and *Pten*, both singly and in combination (8, 9). *Pten-KO* mice develop thyroid adenomas, whereas *Prkar1a-KO* mice develop locally invasive FTC by 1 year of age in around 45% cases (9). In the double KO mice, thyroid cancers were observed in 100% of mice by 2 months of age, and about 1/3 of the mice developed well-differentiated lung metastasis, mimicking the human disease (8).

Of the many alterations that occur in a cancer cell leading to uncontrolled growth, metabolic changes are important ones. Recent work has started to characterize many of these metabolic changes (10–13). We have previously shown that *Prkar1a-KO* and the double KO mice exhibit activation of the mechanistic Target of Rapamycin (mTOR), with higher levels in the double KO animals (8). Similar findings were made in human FTC (8), and have been previously reported in PTC, particularly associated with *BRAF* mutations (14). mTOR is an important regulator of cellular metabolism, cellular growth and proliferation (15), and is an important player in promoting the metabolic changes that occur during tumorigenesis. mTOR is regulated by the AMP-dependent protein kinase (AMPK), which is activated in conditions of nutrition restriction or increased AMP/ATP ratio to enhance energy production (16). Activation of AMPK typically leads to suppression of mTOR activity through phosphorylation of the TSC complex as well as Raptor (17, 18).

There is, however, emerging evidence that AMPK activation can be decoupled from the mTOR pathway (19). These observations suggest that under certain conditions, AMPK may act as a tumor promoter rather than a tumor suppressor (20, 21). Thus, AMPK may serve a context-dependent function, in which the outcome from increased/diminished signaling may depend on tissue type and the presence of other intracellular signals. Although AMPK has been reported as elevated in PTC (22), its role in mediating cell growth *in vitro* is unclear, as studies in rat or human thyroid cell lines have yielded contradictory results (23, 24). Analysis of FTC tumors has not been described.

In this manuscript, we report that AMPK is activated in both mouse and human FTCs, which have concurrent activation of the mTOR pathway. Using mouse models and *in vitro* studies, we determined that LKB1, the tumor suppressor which causes Peutz-Jeghers syndrome (25, 26), mediates signaling from PKA to AMPK. These data suggest that not only does AMPK have context-specific function in modulating the behavior of thyroid tumors, but that LKB1 shares this complex role in the thyroid.

## Materials and Methods

### Mice and Tumor Cell Lines

Mice were housed in University Laboratory Animal Resources maintained vivarium at the Ohio State University. *Prkar1a*^*loxP*/*loxP*^, *Pten*^*loxP*/*loxP*^, and *Thyroid Peroxidase-cre* (*TPO-cre*) mice have been previously described (27–29). All mice were of mixed background, with predominant contributions of 129/SvJ and FVB/N. All animal studies were carried out with approval of the Ohio State University Institutional Animal Care and Use Committee under animal research protocol 2009A0084.

### Immunohistochemistry

Tumors sections were processed and stained as described previously (30) with the following antibodies: p-ACC (#3661-Cell signaling), p-P70S6K1 (#ab129230 Abcam), p-LKB1 (S-428) (#ab138386-Abcam), LKB1 (#ab185734 Abcam), STRAD-α (#ab192789 Abcam) p-CAMKII (#ab32678 Abcam), HADHA (#PA5-27348 Thermo Scientific), HSD17β4 (#OAGA1102-Aviva systems biology). For mouse analyses, thyroid sections from 3-5 independent mouse tumors from each genotype were analyzed, with consistent findings observed within each group.

### Maintenance of Cell Lines

The FTC-133 cell line was maintained in high glucose DMEM-10% FCS, supplemented with 25 mM Hepes buffer, 100 U/ml penicillin, 100 μg/ml streptomycin, 2 mM L-glutamine.

### Western Blot Analysis and Immunoprecipitations

**Figure 1:**
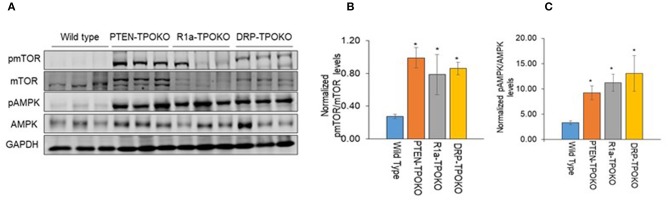
*DRP-TPOKO* and *R1a-TPOKO* tumors show mTOR and AMPK activation. **(A)** Total protein lysates of wild type, *PTEN-TPOKO, R1a-TPOKO*, and *DRP-TPOKO* were run on an SDS gel and immunoblotted for the indicated proteins. GAPDH is used as a loading control. **(B,C)** Western blot analysis of phospho and total mTOR and AMPK was done, which was further normalized to GAPDH. Data is represented as mean± S.E. ^*^p < 0.05.

Cell lysis and Immunoblotting was performed as described previously (8). 30 μg of protein was loaded per lane in 8–10% SDS gels. The following antibodies were used to probe the membranes: p-ACC (S-79) (#11818 Cell signaling), p-mTOR (S-2448) (#ab109268 Abcam), mTOR (#ab2732 Abcam), p-LKB1 (S-428) (#3482 Cell signaling), LKB1 (#3050 Cell signaling), p-AMPK (T-172) (#2535 Cell signaling), p-AMPK (S-173) (#ab55886 Abcam), GAPDH (#2118 Cell signaling), Vinculin (sc-5573, Santa Cruz Biotechnologies), p-TAK1 (#4508 Cell signaling), TAK1 (#5206 Cell signaling), p-CAMKII (#12716 Cell Signaling). For Figures 1, 5, Western blots were developed using near-IR-labeled secondary antibodies and quantitated on a Li-COR Odyssey CLx and analyzed using ImageStudio v5.2. Other blots were developed on film and analyzed semi-quantitatively. All *in vitro* experiments were repeated 2–4 times with consistent results observed.

### Statistical Analysis

Quantitative western blots (Figures 1, 5) were analyzed by one-way ANOVA, followed by *post-hoc* Holm-Sidak multiple comparison test using GraphPad Prism software.

### SiRNA Knockdown

For LKB1 knockdown, smartpool siRNA (On-target plus smartpool, Dharmacon) was used following the manufacturer's recommendations. Briefly, cells were transfected with a 5 μM solution of LKB1 siRNA using Lipofectamine in Optimem (Thermo-Fisher). The cells were treated for 48 h, following which the solution was replaced with DMEM with 10% FBS and treated with PKA activator and/or inhibitor for either 1 or 48 h. Total protein lysate was collected and analyzed by western blotting as above.

### Analysis of Human Thyroid Samples

Paraffin-embedded thyroid tissue samples from patients with thyroid were selected from the thyroid tumor bank maintained under an IRB-approved protocol at the Uniformed Services University of the Health Sciences (USUHS, Bethesda, MD). The tumor bank specimens were obtained as extra material from clinical thyroidectomies and were stripped of identifying information prior to banking; the research therefore is considered exempt and the USUHS IRB waived the requirement for written informed consent for this study.

Histological typing was performed by an experienced pathology (VV) based on World Health Organization criteria. Genetic data for these samples was not considered in choosing thyroid specimens Human tissues for analysis were fixed in neutral buffered formalin and then embedded in paraffin. Sections (4 mm) were dewaxed, rehydrated through graded ethanol, and antigen retrieval was performed in 10 mmol/L citrate buffer, pH 6.0, for 10 min. The sections were cooled and incubated with 3% hydrogen peroxide for 15 min. Sections were next incubated with blocking solution (Vector Labs) for 10 min, followed by incubation with primary antibodies overnight at 4°C. Primary antibody binding was detected with a biotinylated secondary antibody (Vector Labs) followed by a streptavidin-horseradish peroxidase conjugate. Immunoreactivity was revealed with 3,3-diaminobenzidine (Vector Labs). Sections were counterstained with hematoxylin. Every tumor was given a score according to the intensity of the staining and the extent of stained tumor areas (0% score 0; 1–10% score 1; 11–50% score 2; and 51–100% score 3.

## Results

### AMPK and mTOR Activation in Mouse FTC Tumors

We have previously reported that mice with thyroid-specific KO of *Prkar1a* alone *(R1a-*TpoKO) or in combination with *Pten* KO (Double *R1a-Pten KO*, or DRP-TpoKO) exhibit activation of mTOR (8). First, we confirmed this observation in a set of new tumor samples (Figure 1A). To begin understanding the implications of mTOR activation, we next turned to AMPK, which is considered to be a negative regulator of mTOR under most circumstances (31). Surprisingly, when we analyzed thyroid lysates from our mouse models, we found a significant increase in phospho-T172 AMPK, a post-translational modification which leads to protein activation. Activation of AMPK correlated with tumorigenic potential, with minimal increases in WT or non-carcinogenic *Pten* KO, and much higher levels in the carcinogenic R1a- KO and the double KO (Figures 1B,C).

**Figure 2:**
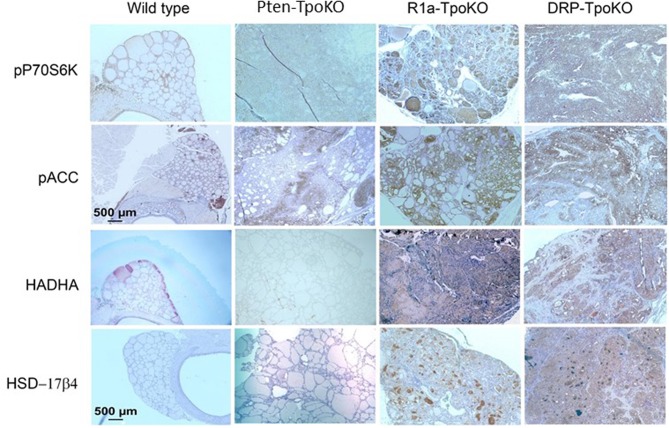
Mouse tumors shown activation of mTOR and AMPK pathways. Representative mouse tumors from the genotypes shown at the top were analyzed by immunohistochemistry for the proteins shown at left. WT, Pten-TpoKO, and R1a-TpoKO mice were 1 year of age, whereas DRP-TpoKO mice were 6 months of age. All photos were taken at the same magnification, with scale bars shown in two panels in the left column.

To confirm dual activation of mTOR and AMPK in the tumors, we studied mouse thyroids sections from 3 to 5 independent mouse tumors from each genotype by immunohistochemistry. We first checked phospho-p70S6K as a marker of mTOR activation, which revealed strong staining in the cancers (Figure 2, top row), as we have previously described (8). To verify AMPK activation, we assessed staining for activated Acyl-coA-Carboxylase (ACC), a downstream target for activated AMPK. This experiment demonstrated diffuse cytoplasmic expression of p-ACC in all tumor mice (Figure 2, second row), with most intense staining intensity observed in more aggressive, invasive and metastatic DKO mice. From a metabolic viewpoint, it has been reported that AMPK activation enhances fatty acid oxidation (FAO) which helps expand energy metabolites for growing tumor and promotes tumor growth. To examine the activation of FAO pathway in mouse tumors, we examined the expression of two enzymes involved in FAO pathway: the mitochondrial protein HADHA (Hydroxyacyl-CoA Dehydrogenase/3-Ketoacyl-CoA Thiolase/Enoyl-CoA Hydratase, Alpha subunit, also known as mitochondrial trifunctional protein), and the peroxisomal protein HSD17B4 (hydroxysteroid 17-beta dehydrogenase 4) (Figure 2, bottom rows). We found overexpression of both of these enzymes in R1a-KO and DKO mice but not in Pten-KO or wild type mice, supporting a functional role for AMPK in altering tumor metabolism. These data indicate that these mouse models of FTC in which PKA is activated exhibit activation of both AMPK and mTOR.

### AMPK Activation Is Mediated by LKB1

To understand the mechanism by which AMPK is activated in thyroid cancer, we turned our attention to kinases known to phosphorylate (activate) AMPK downstream of PKA. There are three major kinases responsible for AMPK phosphorylation: LKB1, CamKII, and TAK1 (19). Mining of our microarray data demonstrated that expression of these kinases was not altered in the tumors, although there were some shifts in isoform expression for CAMKII, with decreases in Camk2a and b, and increases in Camk2d (data not shown). To address alterations in signaling, we used the human FTC cell line FTC-133 to probe the activation of these kinases at baseline and in response to PKA activation.

**Figure 3:**
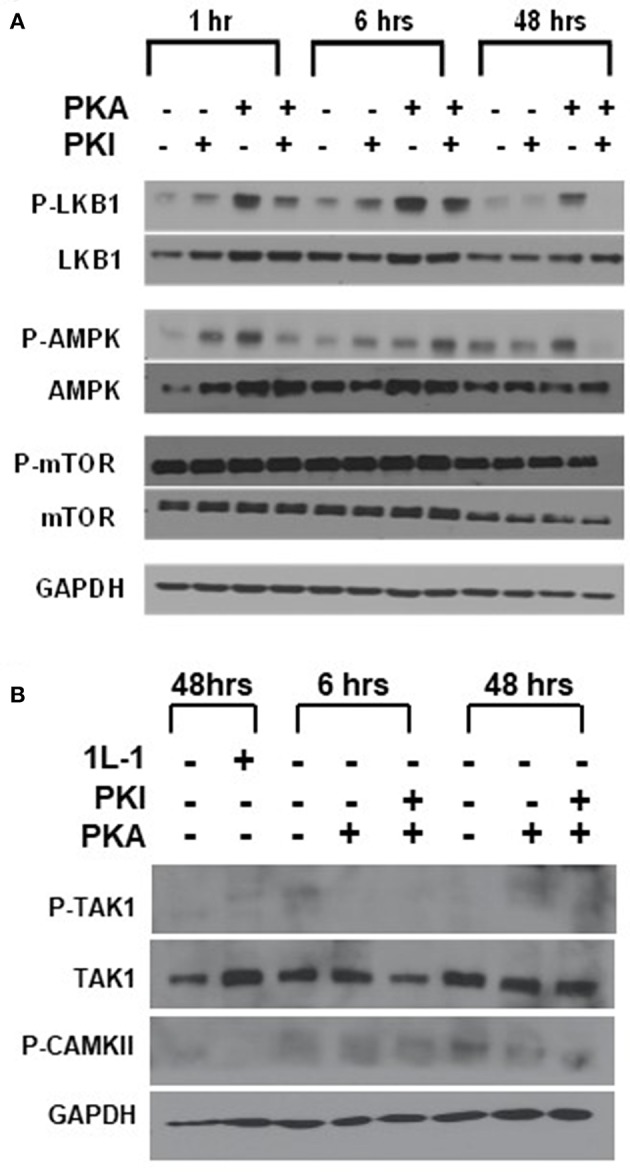
LKB1 is responsible for PKA-mediated stimulation of AMPK. **(A)** FTC-133 cells were treated with PKA activator (PKA) and/ or inhibitor (PKI) for the times shown, and immunoblotted for total and phospho- isoforms of LKB1, AMPK, and mTOR. Note that pLKB1 and pAMPK increase after PKA activator and this is blocked by PKI. **(B)** FTC-133 were treated as above and probed for total or phospho-TAK1 and for pCAMKII. IL-1 treatment is used as a positive control for pTAK1 induction, although induction is very modest in these cells. Note that neither protein is activated in response to PKA. GAPDH is shown as a loading control.

We first examined LKB1, which has been shown to function as a tumor suppressor, as inactivating mutations in the gene cause the inherited tumor predisposition Peutz-Jeghers syndrome (25, 26). Although there are rare cases of *LKB1* mutations in thyroid cancer (32), thyroid cancer's association with this syndrome remains unclear (33, 34). Loss of the gene is also a common somatic event in a subset of patients with non-small cell lung cancer, melanoma, and cervical cancer (35), but activating mutations have not been described. LKB1 is also known to be a substrate for PKA phosphorylation, with this phosphorylation promoting LKB1 activity (36). To explore the role of LKB1 in activating AMP, we measured both LKB1 and AMPK phosphorylation in FTC-133 cells after manipulation of PKA activity (Figure 3A). Treatment of cells with the specific PKA activator 6-Bnz-cAMP led to an increase in both pLKB1 and pAMPK. Treatment of cells with the peptide inhibitor of PKA (PKI) blocked this effect. Despite the activation of AMPK there was no change to p-mTOR phosphorylation levels in response to these manipulations, confirming our underlying observation that AMPK and mTOR activation are decoupled in this system.

We also examined the activation of CAMKII and TAK1 using this same system, as both of these kinases may also be activated by PKA (37, 38). Although we observed CAMKII and p-CAMKII in the cells, levels were not enhanced in response to PKA activation (Figure 3B). Levels of TAK1 were very low in the FTC cells, and there was also no increase in p-TAK1 after activation of PKA. (Figure 3B) These data suggested that PKA activates AMPK through LKB1.

**Figure 4:**
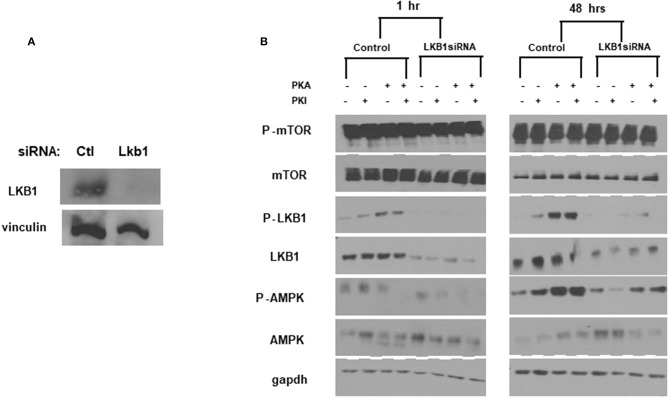
*LKB1* knockdown suppresses PKA-mediated AMPK activation. **(A)** *LKB1*-specific siRNA pool causes knockdown of LKB1 in FTC−133 cells. Non-targeting siRNA was used as a control. Proteins were analyzed 48 h after transfection. Vinculin is shown as a loading control. **(B)** Cells treated with control- or *LKB1*-specific siRNAs were treated with PKA activator (PKA) and/or inhibitor (PKI) for the indicated times and probed for activation (phosphorylation) of mTOR, LKB1, and AMPK. GAPDH was used as a loading control.

To further confirm the ability of PKA to activate AMPK through LKB1, we used siRNA to generate transient knockdown (KD) of LKB1 in FTC-133 cells. The siRNA was consistently able to achieve >80% of the target protein (Figure 4A). After LKB1 KD, the cells were treated with PKA activator and inhibitor to examine AMPK activation. LKB1 knockdown at both 1 and 48 h led to a significant reduction of AMPK activation (Figure 4B) suggesting that LKB1 is the major kinase responsible for AMPK activation downstream of PKA. Note that AMPK phosphorylation was not completely abrogated by LKB1 knockdown, suggesting that either residual LKB1 may suffice to partly activate AMPK, or that other kinases may also play a role in this process. AMPK may also be phosphorylated on S173, which is an inactivating modification of AMPK (39). Although PKA activation has been proposed to cause phosphorylation at this site in hepatocytes (39, 40), we did not observe a band corresponding to AMPK-pS173 under any of our treatment conditions (data not shown).

### LKB1 Activation in Mouse Models of FTC Tumors

**Figure 5:**
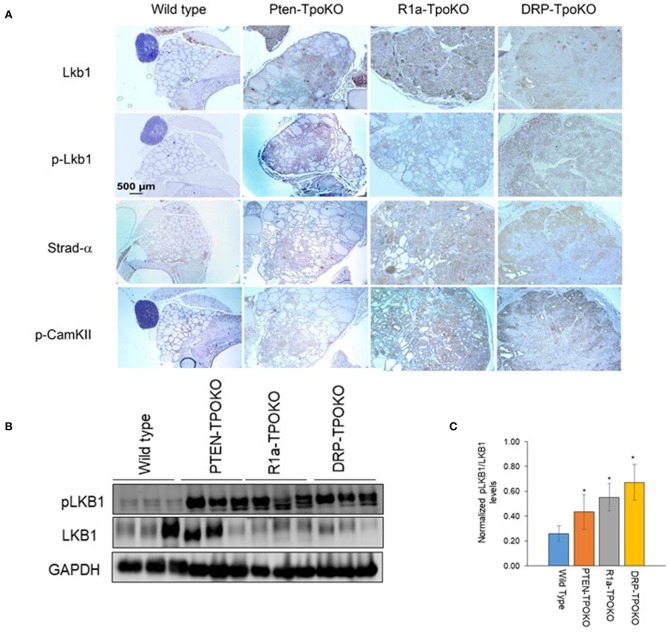
LKB1 is activated in mouse thyroid tumors. **(A)**. Representative immunohistochemical images of wild type, *Pten-, R1a-*, and *DRP-TpoKO* tumors stained for the proteins shown at left. All images were taken at the same magnification corresponding to the scale bar shown in the left column. **(B)** Western blotting of tumor lysates for pLKB1 and total LKB1. GAPDH is shown as a loading control. **(C)** Quantitation of data in panel B. Data is represented as mean± S.E. ^*^p < 0.05.

In order to make sure that these observations were relevant *in vivo*, we next sought to assess LKB1 activation in mouse tumors (Figure 5A). In WT thyroids, there was little staining of either total or pLKB1 in the tissue. For the tumor models, total and pLKB1 were increased in *Pten* and *R1a* knockouts, and further increased in the double KO. These observations were confirmed by western blot for pLKB1 from tumor lysates (Figure 5B). As LKB1 function is partly dependent on nuclear-cytoplasmic shuttling mediated by the pseudosubstrate Strad-α (41), we also performed IHC for this protein. Our results demonstrate that Strad-α is not expressed in WT thyroids or *Pten* tumors, but exhibits strong staining in the *R1a*-KO and DKO tumors. Taken together, our results suggest that PKA activates the LKB1-AMPK pathway in PKA-driven mouse models of FTC, including both locally aggressive and metastatic disease.

Since activation of CAMKII was also observed *in vitro* (Figure 3B), we assessed its expression in our mouse tumors. We found increased expression of p-CAMKII in *R1a-KO* and DKO tumors compared to that from *Pten*-KO and wild type thyroids (Figure 5A, bottom row). Interestingly, although p-CAMKII expression was uniform in the R1a KO tumors, expression in the DKO tumors showed regional differences, with much darker staining in the outer edges of the tumor compared to its central region. This expression pattern in the DKO tumors did not correlate with the patterns of AMPK or mTOR activation (Figure 2).

### Lack of Inhibition of CREB Pathway by LKB1 and Activation of AMPK Related Kinases

**Figure 6:**
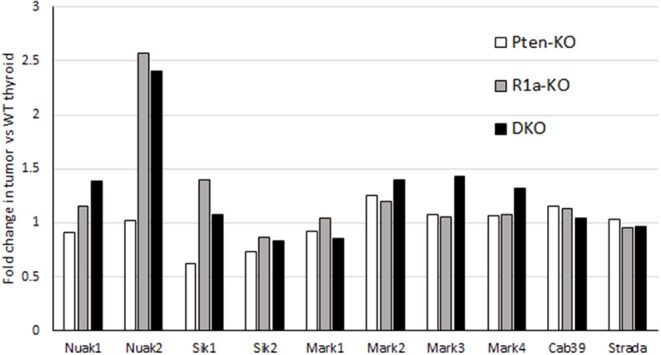
mRNA expression of LKB1 binding partners and AMPK-like kinases in mouse models of FTC. For each model, mRNA expression is expressed as fold-change compared to WT littermates. Data is collected from Pringle et al. (8).

LKB1 not only activates AMPK but 13 other AMPK-like kinases. To assess the expression levels of these AMPK-like kinases, we mined data from our previous microarray analysis of tumors (8). We found no loss of expression of any of the 8 AMPK-related kinases or LKB1 binding partners including CAB39 (MO25) or Strad-α (Figure 6) (42). Our data does show preferential upregulation of the expression of NUAK2 and SIK1 in *R1a* and DKO tumors, although we did not perform measurements of the activity of these kinases. In lung cancer, the functional significance of LKB1 loss has been assessed, and it was demonstrated that LKB1 activation inhibits a set of 200 genes which were defined as a CREB-dependent cluster (43). Examination of this gene set demonstrated no significant change in the *R1a* and DKO mice, suggesting that LKB1 does not inhibit the CREB-mTOR pathway in our mouse model of FTC (data not shown).

### LKB1-AMPK Pathway in Human Thyroid Cancer

**Figure 7:**
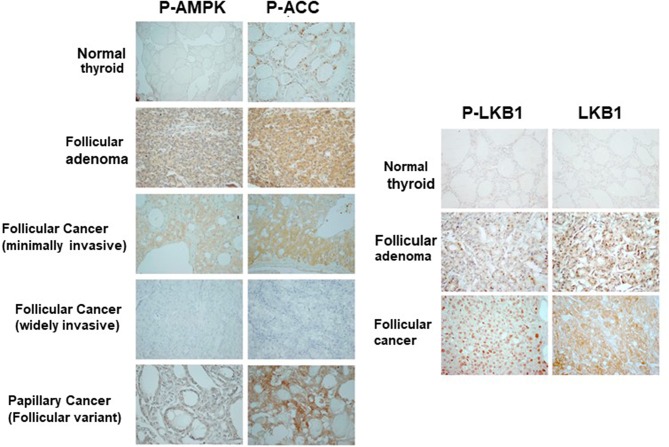
Activation of AMPK and LKB1 in human thyroid neoplasias. Representative immunohistochemical images of tissue sections from patients with benign and malignant follicular thyroid lesions stained for p-AMPK, p-ACC, and pLKB1. These data are quantitated in Tables 1–3.

So far, our data suggests that PKA activates the LKB1-AMPK pathway in our mouse models of FTC. However, we wanted to assess the activation of this pathway in human follicular thyroid lesions. Phospho-AMPK staining (Figure 7, Table 1) was detected in thyrocytes in 15% (3/20) of benign follicular adenomas with rates that were slightly higher for FV-PTC (20%) and FTC (30%), although these numbers are not large enough to say if these modest differences are significant. The overall intensity of staining assessed on a graded scale was highest in FTCs, but again small numbers preclude any type of statistical conclusion. We did note that less-differentiated and widely invasive FTCs did not stain for p-AMPK. As in the mouse models, p-AMPK staining in human tumors generally correlated with activation of its downstream target p-ACC (Table 2), although p-ACC staining was much more common, including ready detection in the normal thyroid gland.

**Table 1:** p-AMPK expression in human follicular thyroid lesions.

**Histology**	**# of cases**	**pAMPK IHC score**			**% Low staining (1)**	**% High staining (2)**
		**0**	**1**	**2**		
Normal thyroid	20	18	2	0	10	0
Follicular adenoma	20	17	2	1	10	5
Follicular cancer	20	14	4	2	20	10
Follilcular variant of papillary thyroid cancer	20	16	4	0	20	20

**Table 2:** p-ACC expression in human follicular thyroid lesions.

**Histology**	**# of cases**	**pAMPK IHC score**			**% Low staining (1)**	**% High staining (2)**
		**0**	**1**	**2**		
Normal thyroid	20	10	9	1	45	5
Follicular adenoma	20	8	8	4	40	20
Follicular cancer	20	12	4	4	20	20
Follilcular variant of papillary thyroid cancer	20	8	8	4	40	20

Using a different cohort of human samples, we also assessed total and phospho-LKB1 staining in the thyroid (Figure 7 right, Table 3). In the wild type gland, LKB1 was detected in half the cases (5/10), but was rarely phosphorylated (1/10 cases). Follicular adenomas exhibited light staining for total and p-LKB in about half the cases (4/8), with another 25% lacking staining. In contrast, there was much more prominent activation of this kinase in malignant lesions, with 6/12 PTCs showing heavy staining for both protein forms (total and phosphorylated). Further, all FTCs stained for total LKB1, with 9/13 (69%) showing intense staining for the total protein and the large majority of tumors (10/13, 77%) showing high level phosphorylation of LKB1. Of note, immunoactive LKB1 was greatest in regions of capsular invasion; and was localized to the nucleus in follicular as well as in papillary cancer cells. The highest intensity staining was observed in tumors characterized as Hurthle cell cancers (data not shown). We have previously reported that human FTCs exhibit strong nuclear staining for phospho-CREB as a marker of PKA activation (8). These observations suggest that the PKA-LKB1 axis is active in human FTCs as well as those from our mouse model.

**Table 3:** LKB expression in human thyroid tumors.

**Histology**	**# of cases**	**LKB IHC score**			**% High staining (2)**	**pLKB IHC score**			**% High staining (2)**
		**0**	**1**	**2**		**0**	**1**	**2**	
Normal thyroid	10	5	4	1	10	7	2	1	10
Follicular adenoma	8	2	4	2	25	2	4	2	25
Follicular cancer	13	0	4	9	69	1	2	10	77
Papillary thyroid cancer	12	3	3	6	50	2	4	3	25

## Discussion

Carney complex (CNC) is a tumor predisposition syndrome which includes thyroid neoplasia in about 25% of patients, including 2.5% of patients who develop frank thyroid carcinoma (44), including a predominance of the FTC subtype. It has been demonstrated that CNC is caused by inactivating mutations in *PRKAR1A* gene, which is a regulatory subunit of PKA (6), suggesting that dysregulation of PKA signaling can cause tumor formation in the thyroid gland, and in other affected tissues. We have previously demonstrated that activation of PKA is the main driver of tumorigenesis and other phenotypes related to KO of *Prkar1a*, as genetic reduction of PKA catalytic subunits suppresses these phenotypes (45). However, the exact signaling pathways activated by PKA which cause tumor formation have not been elucidated in detail. Furthermore, our previous publication demonstrated that PKA-mediated tumors do not involve typical signaling pathways such as AKT and ERK activation (9). Therefore, our present endeavor was to understand the PKA signaling and understand the molecular signaling pathways involved in PKA-mediated FTC tumors.

The role of AMPK in cancer development is controversial with conventional wisdom terming it a tumor suppressor (17). However, evidence in the recent literature points to a more nuanced context-dependent role, which may include activity as either a tumor promoter or a suppressor (46). In many cancers, AMPK has been shown to negatively regulate the mTOR pathway through TSC-2 and Raptor and dephosphorylation of PRAS40 (47, 48). However, our mouse tumors and also *in vitro* data from FTC-133 cells show an absence of negative regulation of mTOR pathway despite AMPK activation. Some authors have also concluded that activated AMPK and mTOR can coexist together without affecting each other (19). Other research has shown that AMPK may not be completely sufficient to inhibit the mTOR pathway, and AMPK inactivation does not necessarily activate mTOR pathway (19). Of particular interest to us was the observation that AMPK was activated in R1a-KO and DKO mice suggesting that this pathway must involve PKA signaling. Of the major AMPK kinases responsible for activation of AMPK, LKB1 and CAMKII both have been shown in the literature to be activated by PKA (37, 49). Therefore, we decided to explore whether PKA could be signaling through either of these kinases to activate AMPK.

The serine-threonine kinase LKB1 (STK11) is mutated in the tumor predisposition Peutz-Jeghers syndrome (PJS), an inherited human disorder characterized by skin pigmentation and hamartomatous polyps of the GI tract (50). Because PJS mutations are inactivating, *LKB1* has been termed as a tumor suppressor gene, and its loss is associated with lung cancer, melanoma, and cervical cancer (35). It has been suggested that PJS patients may be associated with an enhanced risk for thyroid cancer (51), but larger studies of this patient population have failed to confirm a clear predisposition (33, 34). In sporadic thyroid cancers, LKB1 has not been well studied, although two recent publications have reported a total of 3 *LKB1* mutants in thyroid cancer, including a frameshift, a splice site mutation, and a missense mutation (32, 52). The functional significance of these mutants needs to be characterized, but this data points to that fact that *LKB1* mutations are rare in thyroid cancer.

LKB1 is known to inhibit the mTOR pathway through activation of AMPK, which may inhibit tumor cell proliferation, inhibit tumor cell polarization, and inhibit tumor metastasis (42). In many tumors therefore, LKB1 expression has been negatively associated with increased aggressiveness of the tumor (42), observations which have also been confirmed in mouse models and using *in vitro* systems (53, 54). In *LKB1* heterozygous mice, there was no loss of LKB1 expression observed from the gastrointestinal polyps, suggesting initiation of polyp formation may not be due to LKB1 loss (54). These observations have led authors to suggest a context-dependent function of LKB1, where loss in early tumorigenesis leads to the formation of benign tumors whereas its loss in late tumorigenesis induces the formation of malignant tumors (53).

Our interest in examining the role of LKB1 arose from the fact that LKB1 is known to be a direct substrate of PKA (36). Our tumors demonstrated robust total LKB1 expression which was diffuse and cytoplasmic or membranous. A recent article examining LKB1 expression in breast cancer has suggested that the cytoplasmic localization of LKB1 was associated with worse prognosis (55). Of particular interest was the role of phosphorylation of LKB1 at Serine 428 (Ser428). This particular site has been known to be a target of p90RSK and PKA (36, 49). Ser428 has been reported to inhibit the basal phosphorylation of AMPK but seems to be required for inducible phosphorylation (49). Data in this manuscript indicates, at least in the thyroid, that AMPK is activated in response to LKB1 Ser428 phosphorylation by PKA. Further, activation of AMPK by this signaling cascade does not inhibit mTOR activation in our mouse tumors, an FTC cell line, and a subset of human thyroid tumors (which show high AMPK pathway activation). This observation also suggests the presence of additional regulatory proteins which prevent AMPK from inhibiting mTOR pathway. Recently there is increasing evidence suggesting that LKB1 subcellular location is regulated by various proteins and nuclear receptors, such as PKC-zeta (56), ROR-alpha, estrogen receptor, Farnesoid X receptor, and Nur77 orphan nuclear receptor (57). Nur77, another well-known PKA target (58, 59), has been shown to directly bind to LKB1 and shuttle it to nucleus and thus modulate AMPK activation levels in tissues (57). However, in our tumors we did not find any evidence of nuclear localization of Nur77, suggesting that this protein might not be responsible for nuclear localization of p-LKB1 in our tumor models (data not shown).

LKB1 in recent years has been linked to activation and phosphorylation of 13 AMPK- related kinases, such as the MARK, SIK, and NUAK family of proteins (42). In general, these kinases have been termed as tumor promoters/suppressors, depending on the tumor type and context. For instance, NUAK2 has been shown as a tumor promoter in different tumor models (60–63). SIK-1, -2, and -3 have been demonstrated to be acting as tumor suppressors and also tumor promoters; the latest example being of SIK2 which was shown to be acting as a metastasis promoter in ovarian cancer (64). Similarly, the MARK family of proteins have also been implicated to aid in invasion and aggressiveness of tumor growth (46, 65). Although there are various pathways implicated in overexpression of these proteins, they all seem to require active LKB1 (42). Indeed, the fact that human FTCs exhibited frequent LKB1 activation, even when AMPK was not present, suggests that LKB1 may signal through multiple pathways to promote tumor formation and/or progression. These observations suggest that LKB1 can act as tumor promoter/suppressor through various pathways promoting/suppressing metabolism, aggressiveness, invasion and metastasis.

In summary, our combined *in vitro-in vivo* study clearly demonstrates that FTCs can have concomitant activation of both the mTOR and AMPK pathways. In PKA-driven tumors, PKA activates AMPK through the LKB1 kinase. Although LKB1 has generally been considered to be a tumor suppressor, our data indicates that its role in the thyroid can be more complex, with the protein serving as mediator downstream of PKA activation in driving tumorigenesis. Like AMPK, LKB1 appears to serve as a context-dependent tumor promoter, and activation of this signaling pathway can be decoupled from regulation of mTOR signaling. Our ongoing studies continue to be focused around elucidating the details of these steps in the process of tumor formation and tumor growth.

## Data Availability Statement

All datasets generated for this study are included in the article/supplementary material.

## Ethics Statement

Ethical review and approval was not required for the study on human participants in accordance with the local legislation and institutional requirements. Written informed consent for participation was not required for this study in accordance with the national legislation and the institutional requirements. The animal study was reviewed and approved by The Ohio State University Institutional Animal Care and Use Committee.

## Author Contributions

SK performed and analyzed experiments for this study, and wrote and edited the manuscript. VV performed all analysis of human pathology specimens and reviewed the manuscript. SP performed and analyzed additional experiments for the study, and edited the manuscript. LK designed, supervised, and analyzed the work, and wrote and edited the manuscript.

### Conflict of Interest

The authors declare that the research was conducted in the absence of any commercial or financial relationships that could be construed as a potential conflict of interest.
